# Do Lipids Influence Gastrointestinal Processing: A Case Study of Major Soybean Allergen Gly m 4

**DOI:** 10.3390/membranes11100754

**Published:** 2021-09-30

**Authors:** Ekaterina I. Finkina, Daria N. Melnikova, Ivan V. Bogdanov, Anastasia A. Ignatova, Tatiana V. Ovchinnikova

**Affiliations:** 1 M. M. Shemyakin & Yu. A. Ovchinnikov Institute of Bioorganic Chemistry, The Russian Academy of Sciences, Miklukho-Maklaya str., 16/10, 117997 Moscow, Russia; d_n_m_@mail.ru (D.N.M.); contraton@mail.ru (I.V.B.); aignatova_83@mail.ru (A.A.I.); ovch@ibch.ru (T.V.O.); 2 Department of Biotechnology, I. M. Sechenov First Moscow State Medical University, Trubetskaya str., 8–2, 119991 Moscow, Russia

**Keywords:** Bet v 1 homologue, Gly m 4, soybean allergen, lipid binding, pepsin, gastric digestion

## Abstract

Previously, we have demonstrated that Gly m 4, one of the major soybean allergens, could pass through the Caco-2 epithelial barrier and have proposed a mechanism of sensitization. However, it is not known yet whether Gly m 4 can reach the intestine in its intact form after digestion in stomach. In the present work, we studied an influence of various factors including lipids (fatty acids and lysolipids) on digestibility of Gly m 4. Using fluorescent and CD spectroscopies, we showed that Gly m 4 interacted with oleic acid and LPPG (lyso-palmitoyl phosphatidylglycerol), but its binding affinity greatly decreased under acidic conditions, probably due to the protein denaturation. The mimicking of gastric digestion revealed that Gly m 4 digestibility could be significantly reduced with the change of pH value and pepsin-to-allergen ratio, as well as by the presence of LPPG. We suggested that the protective effect of LPPG was unlikely associated with the allergen binding, but rather connected to the pepsin inhibition due to the lipid interaction with its catalytic site. As a result, we assumed that, under certain conditions, the intact Gly m 4 might be able to reach the human intestine and thereby could be responsible for allergic sensitization.

## 1. Introduction

Soybean (*Glycine max*) is a widespread protein-rich legume, which is commonly used as a meat substitute and a food additive. The prevalence of soybean allergy in East Asia is considered to be linked with the critical importance of soybeans for traditional Asian cuisine [1]. Clinically relevant immediate-type and late-phase allergic reactions may occur in atopic children younger than 3 years old when milk is substituted by soybean products in patients with cow’s milk allergy [2]. Soybean can cause both mild local and severe systemic allergic reactions [3].

Gly m 4 is one of the most important soybean allergens, which belongs to the Bet v 1 homologue family and is present in soybean seeds at relatively high levels (0.36–0.6 mg/g of seeds) [4]. Bet v 1 homologues share similar spatial structure and are characterized by having an internal hydrophobic cavity, which is able to bind a broad spectrum of ligands, including fatty acids, cytokinins, flavonoids, and so on [5]. At the same time, nothing is known about physiological ligands of the Gly m 4 allergen. Similar to other Bet v 1 homologues, Gly m 4 is an allergen cross-reacting with the major sensitizer of this family, the birch pollen Bet v 1, and is responsible for pollen-related soybean allergy in the Central and Northern Europe [6]. Allergic reactions, often taking place after consumption of moderately processed soybean milk, are most likely provoked by a high Gly m 4 content [3]. In contrast to many other Bet v 1-like food allergens, Gly m 4 can cause severe allergic reactions including anaphylaxis after consuming soybean-containing food [7]. However, a matter for severe allergic reactions in case of Gly m 4, compared with other Bet v 1 homologues, is still unknown.

One of the key features of true food allergens is their resistance to cleavage by proteolytic enzymes in the human gastrointestinal tract. Intact proteins and their fragments at microgram level can reach the intestinal wall, be absorbed, and cause sensitization of the immune system [8]. Therefore, different factors affecting protein uptake and digestibility may influence their allergenicity [9,10]. Such factors include but are not limited to the following: abundance of the allergen in a total protein fraction; variation of pH values due to the buffer capacity of the ingested food; health conditions and incidence of gastrointestinal diseases; and impact of food matrix, such as lipids and carbohydrates. Lipids are nutrient substances and components of gut secretion fluids. At high concentrations, they form micelles and liposomes, which may adsorb and insert proteins due to polar and hydrophobic interactions, protecting them against proteases. On the other hand, lipids may induce protein denaturation and subsequent proteolysis [11,12]. At the same time, at low concentrations, lipid molecules in themselves may possess high biological activity and affect an action of proteolytic enzymes [13]. Besides, some allergens, including Bet v 1 homologues, bind lipid ligands, which leads to changes in their structure and sensitivity to proteolysis [14].

Previously, we have shown that the soybean allergen Gly m 4 is susceptible to the pepsin cleavage. At the same time, we have demonstrated that not only proteolytic fragments but also the Gly m 4 intact form are able to pass through the Caco-2 epithelial barrier and induce the production of Th2-associated cytokines by immunocompetent cells [15]. The main goal of this work was to elucidate whether Gly m 4 can reach the intestinal epithelium in its intact form. With this end in view, we investigated the effects of pH changes, enzyme loading, and adding of lipid molecules (fatty acids (FAs) and lysolipids), which are present in soybean and other food products and can bind to Gly m 4, on the structure and digestion of the soybean allergen in human stomach. In addition, using computer simulation and fluorescent spectroscopy, we studied an influence of lipid molecules on the gastric enzyme pepsin properties.

## 2. Materials and Methods

### 2.1. Materials

Synthetic lysophospholipids were purchased from Avanti Polar Lipids (Alabaster, AL, USA). FAs and 2-p-toluidinonaphthalene-6-sulphonate (TNS) were purchased from Sigma-Aldrich (St. Louis, MO, USA). Digestive enzymes (porcine pepsin and trypsin, bovine α-chymotrypsin) as well as model protein substrates (bovine α-casein and cytochrome *c*) were purchased from Sigma-Aldrich. The recombinant Gly m 4 was overexpressed in *E. coli* and purified as described previously [15].

### 2.2. Fluorescence Spectroscopy

Fluorescence measurements were performed at 25 °C with the F-2710 spectrofluorometer (Hitachi High Technologies America Inc., Pleasanton, CA, USA). The excitation and emission wavelengths were set at 320 and 437 nm, respectively. TNS (4 µM) with Gly m 4, α-casein, cytochrome *c* or pepsin (4 µM in each case) was incubated for 2 min in a stirred cuvette containing 2 mL of the 0.01 M phosphate buffer, pH 7.4, 0.05 M sodium acetate, pH 5.0, 0.05 M sodium acetate, pH 3.5, or 0.05 M HCl, pH 2.0, with gentle mixing before the initial fluorescence (F_0_) was recorded. Then, a lipid (4 µM) was added and, 2 min later, the fluorescence was recorded at equilibrium (F). The experiments were performed in triplicate. The results were expressed as a percentage of the protein-TNS complex fluorescence calculated according to the formula [(F−F_0_)/F_C_] × 100%, where F_C_ is the fluorescence of the protein-TNS complex in the absence of a lipid.

### 2.3. Simulation of Gastric Digestion of Soybean Allergen In Vitro

Gly m 4 cleavage mimicking gastric digestion in vitro was performed for 2 h at 37 °C using porcine pepsin. In order to investigate the effects of different pH and the enzyme loading, the pepsin cleavage of Gly m 4 was carried out using 50 ng (0.1 U), 5 ng (0.01 U), or 0.5 ng (0.001 U) of the enzyme per 1 μg of Gly m 4 (enzyme-to-allergen mass ratio 1:20, 1:200, or 1:2000, respectively) in 0.05 M HCl, pH 2.0, or in 0.05 mM sodium acetate, pH 3.5, or in 0.05 mM sodium acetate, pH 5.0. Lipids at final concentrations of 0.02 and 0.2 mM were preincubated with allergen for 10 min in order to examine their impact on the pepsin digestion of Gly m 4. Pepsin cleavage of model proteins, α-casein and cytochrome *c*, in the presence of or without lipids, was also performed in 0.05 M HCl, pH 2.0, for 2 h using 0.5 ng (0.001 U) or 0.1 ng (0.0002 U) of the enzyme per 1 μg of these proteins (enzyme-to-substrate mass ratio 1:2000 or 1:10,000, respectively). Digestion of all the proteins was monitored by sodium dodecyl sulfate polyacrylamide gel electrophoresis (SDS-PAGE) [16]. Each experiment was carried out three times. Gel analysis was performed using Gel Doc XR^+^ imaging system (Bio-Rad, Hercules, CA, USA) and Image Lab Software. Stability of Gly m 4 in sterile solutions with different pH values was studied by incubation of the allergen for a day and analyzed by SDS-PAGE.

### 2.4. CD Spectroscopy

Circular dichroism spectra were recorded at room temperature (25 °C) using a J-810 spectropolarimeter (Jasco, Hachioji, Tokyo, Japan) in a cell with an optical path of 0.01 cm in a wavelength range of 190–250 nm (scan rate 1 nm) using solutions of Gly m 4, pepsin, α-casein, or cytochrome *c* in pure water, pH 6.5, or in 0.05 M sodium acetate, pH 5.0, or in 0.05 M sodium acetate, pH 3.5, or in 0.05 M HCl, pH 2.0, at concentrations of 0.02–0.06 mM. Lysolipids were added at final concentration of 0.2 mM. Each experiment was carried out twice. Solutions of lipids in the different solvents were used as controls.

### 2.5. Bioinformatic Approaches to Studying of Protein-Lipid Interactions

Three-dimensional X-ray crystal structure of porcine pepsin (PDB ID: 4PEP) was obtained from the Protein Data Bank (PDB) and prepared for the molecular docking simulation by using DockPrep tool of the UCSF Chimera v.1.4 software package (San Francisco, CA, USA) [17]. The 3D structures of lauric acid (C12:0, LAU) (PubChem CID: 3893), stearic acid (C18:0, STE) (PubChem CID: 5281), and oleic acid (C18:1, OLE) (PubChem CID: 445639) were obtained from PubChem database (NCBI, USA). The 2D conformers of LPPC (1-palmitoyl-2-hydroxy-sn-glycero-3-phosphocholine) (PubChem CID: 460602) and behenic acid (C22:0, BEH) (PubChem CID: 8215) were obtained from the PubChem database and subsequently converted into 3D space with energy minimization and geometry optimization by Open Babel v3.0.0 software [18]. The 3D molecule of LPPG (1-palmitoyl-2-hydroxy-sn-glycero-3-phosphoglycerol) was obtained from NMR solution structure of Lc-LTP2 complexed with LPPG (PDB ID: 5LQV). The docking box was chosen so that the whole protein molecule in the ribbon representation was entirely inside this box. Blind docking of the ligands into protein molecules was based on the Lamarckian genetic algorithm (LGA) and was performed by AutoDock Vina tool of the UCSF Chimera v.1.4 software [19]. Protein–ligand interactions were visualized and analyzed with Discovery Studio Visualizer [20].

## 3. Results

### 3.1. Lipid-Binding Assay

Little is known about the ability of Gly m 4 to bind lipid ligands. In our previous work, we have shown that the soybean allergen binds to flavonoid quercetin 3,4′-diglucoside with moderate affinity [15]. In this study, such FAs as LAU, STE, BEH, and OLE, as well as two lysolipids, LPPG and LPPC, were used as possible endo- and exogenous ligands for Gly m 4, because they are components of soybean and gastric secretions [21,22].

**Figure 1:**
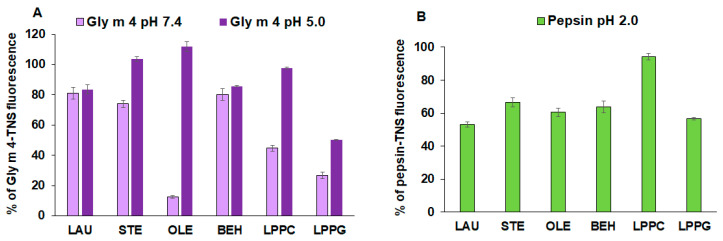
The effect of fatty acids and lysolipids on the fluorescence level of the Gly m 4-TNS (**A**) and pepsin-TNS (**B**) complexes. The results are expressed as the mean values (±SD) of the percentage of fluorescence using the protein-TNS complexes without ligands as controls.

To investigate whether not only Gly m 4, but also the gastric enzyme pepsin and model proteins, α-casein and cytochrome *c*, are able to bind lipid molecules in an aqueous solution, TNS displacement assay was used (Figure 1A,B). TNS is a highly fluorescent molecule when dissolved in a low polarity medium or bound to proteins. Binding of TNS to the Gly m 4, α-casein or pepsin resulted in an increase in fluorescence intensity, the value of which was taken as 100% for each compound. Cytochrome *c* did not bind TNS due to the absence of hydrophobic regions on its surface.

In this work, we showed for the first time the ability of Gly m 4 to bind different lipid ligands. At pH 7.4, low binding affinity of Gly m 4 for saturated fatty acids (LAU, STE, BEH) was observed. By contrast, unsaturated OLE efficiently displaced TNS (12% of the control fluorescence). In the case of LPPC and LPPG, the reduction of the Gly m 4-TNS fluorescence was evident and achieved 44% and 26% of the control fluorescence, respectively (Figure 1A). However, at pH 2.0 and pH 3.5, Gly m 4 did not bind lipids (data not shown). The lipid-binding capacity of the allergen was examined also under less acidic conditions, at pH 5.0. In this case, Gly m 4 did not bind STE and OLE. A very low affinity of the allergen for LPPC was shown (97% of the control fluorescence). Binding of LAU and BEH was similar to those at pH 7.4. At pH 5.0, the decrease in Gly m 4-TNS fluorescence in the presence of LPPG was obvious and reached 51% of the control fluorescence, compared with 26% at pH 7.4.

Experiments with pepsin and model proteins, α-casein and cytochrome *c*, were carried out only under acidic conditions, at pH 2.0. Surprisingly, pepsin bound to the tested FAs and LPPG with an equal efficiency, but almost did not associate with LPPC (94% of the control fluorescence) (Figure 1B). The model protein cytochrome *c*, as expected, did not bind lipids at all. It is known that α-casein belongs to the family of unfolded proteins under native conditions; however, due to the presence of different hydrophobic and hydrophilic regions on its surface, it can interact with other proteins and also with lipid molecules [23,24]. However, in our experiments, α-casein showed very low affinity to all tested lipids, which practically did not displace the TNS from the hydrophobic regions of the protein molecule (data not shown).

### 3.2. Effect of pH and Lipids on Protein Secondary Structures

**Figure 2:**
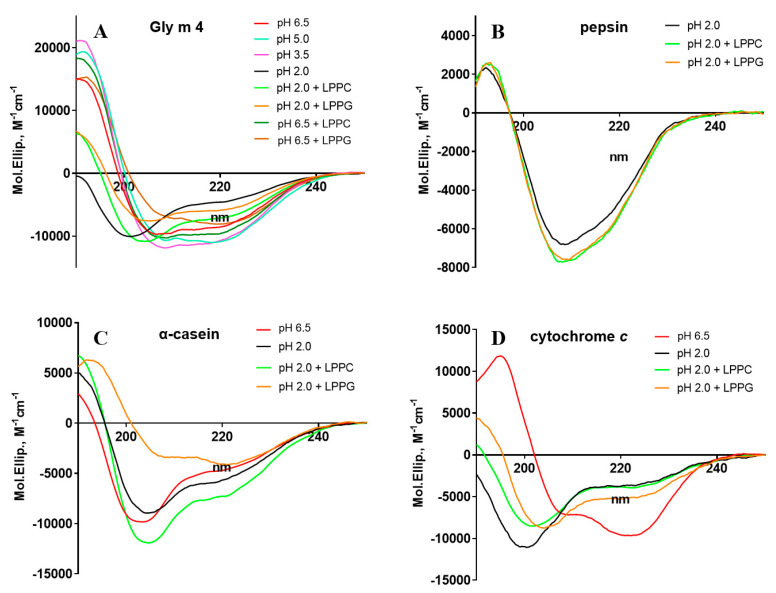
The effects of different pH and lysolipids (at final concentration of 0.2 mM) on the secondary structure of Gly m 4 (**A**), pepsin (**B**), α-casein (**C**), and cytochrome *c* (**D**).

The effects of pH and lipids on protein structures were examined by CD spectroscopy. The far-UV CD spectrum of Gly m 4 at pH 6.5 showed a combination of α- and β-secondary structures characteristic of other Bet v 1 homologues with a positive maximum, at 190 nm, and two negative extremes, at 206 and 222 nm (Figure 2A, Table S1). The CD spectra of Gly m 4 at pH 3.5 as well as at pH 5.0 close to the calculated pI of the protein (4.69) were partially the same but revealed an increase in α-helix content in the last one. By contrast, the CD spectrum of the allergen at pH 2.0 showed denaturation of the protein. The presence of LPPG and, to a lesser extent, LPPC at concentration of 0.2 mM at pH 2.0 and also at pH 6.5 increased the percentage of α-helices in the Gly m 4 structure.

It is known that pepsin predominantly consists of β-sheets and small α-helical segments [25]. The CD spectra of pepsin in the presence of lysolipids or without them at pH 2.0 were the same and had negative extreme at about 208 nm and small positive peak at 192 nm (Figure 2B, Table S1). After the addition of LPPC or LPPG at concentrations of 0.2 mM, the intensity of the negative peak increased slightly, but any significant shifts of the peaks were not observed.

As shown, α-casein from mammalian milk has no well-defined structure but exists in nature as a micellar aggregate [23]. The CD spectrum of α-casein at pH 6.5 showed negative extremes at about 203 and 222 nm (Figure 2C, Table S1). The calculations for the secondary structural elements of the protein at pH 2.0 revealed a slight increase in α-helix content. The presence of lysolipids in the protein solutions at pH 2.0 led to a change in the shape of the CD spectrum indicating conformational rearrangements of the protein structure.

Cytochrome *c* is a small heme protein having predominantly α-heliсal structure [26]. The far-UV CD spectrum of this protein at pH 6.5 showed a positive maximum at 195 nm, typical for α-helical elements, and two negative extremes at 208 and 222 nm (Figure 2D, Table S1). However, as in the case of Gly m 4, at pH 2.0, the protein denaturation was observed. The presence of lysolipids slightly increased the content of α-helixes, which was more pronounced in the presence of LPPG.

### 3.3. Proteolysis by Pepsin Mimicking Gastric Digestion under Different Conditions

**Figure 3:**
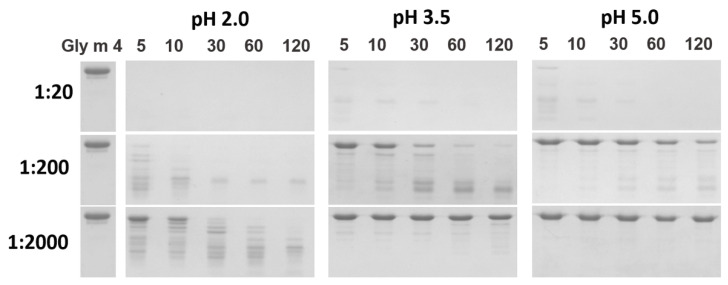
The effects of different pH values and enzyme loading on pepsin digestion of Gly m 4, as determined by SDS-PAGE (Gly m 4—the allergen without pepsin; 5, 10, 30, 60, and 120—digestion time, min; 1:20, 1:200, and 1:2000—pepsin-to-allergen mass ratio).

**Figure 4:**
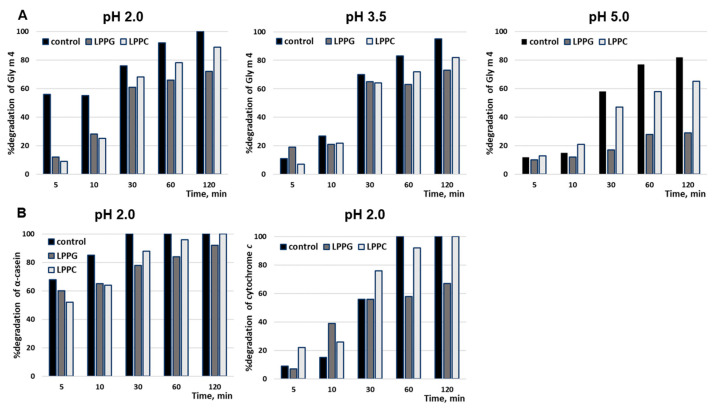
Analysis of pepsin digestion of Gly m 4 and model proteins: (**A**) Gly m 4 at different pH values (enzyme-to-substrate mass ratio of 1:2000 or 1:200 at pH 2.0 or pH 3.5 and pH 5.0, respectively); (**B**) α-casein and cytochrome *c* cleaved at pH 2.0 (enzyme-to-substrate mass ratio of 1:2000 and 1:10,000, respectively). The bands corresponding to intact proteins were registered using Gel Doc XR+ imaging system (Bio-Rad) and Image Lab Software. The percentage of the digested proteins was calculated by formula (So − Sa)/So × 100%, where So is an area of the protein band in a sample without pepsin, and Sa is an area of the protein band at a specified time. Controls—proteins cleaved in the absence of lysolipids.

Earlier, we have shown that the soybean allergen Gly m 4 is highly susceptible to proteolysis [15]. Gly m 4 was almost completely degraded by pepsin with enzyme-to-allergen mass ratio of 1:20 at pH 2.0 in the first 5 min under conditions mimicking its gastric digestion in vitro (Figure 3). Here, we investigated Gly m 4 cleavage by pepsin at less acidic pH and with lower enzyme-to-allergen ratios mimicking its gastric digestions in infants and elderly people as well as in adults after food consumption. The stability of Gly m 4 against gastric digestion was remarkably promoted in the case of simultaneous increase in pH value and decrease in enzyme loading (Figure 3). At pH 3.5 and pepsin-to-allergen ratio of 1:200, the band corresponding to Gly m 4 was observed even after 2 h of incubation, as shown by SDS-PAGE. According to densitometric gel analysis, about 20% of the intact protein was present in hydrolysate after 2 h, at pH 5.0, at the same enzyme-to-substrate ratio (Figure 4). In the case of pepsin-to-Gly m 4 ratio of 1:2000, even at pH 2.0, a trace amount of the soybean allergen was observed after 1 h of the protein digestion. At this enzyme-to-substrate ratio, Gly m 4 particularly was not cleaved at pH 5.0 as well as at pH 3.5. The stability of Gly m 4 in solutions with different pH values was examined (Supplementary Figure S1). Hydrolysis of the protein in the absence of enzyme was observed in solutions with acidic pH values.

**Figure 5:**
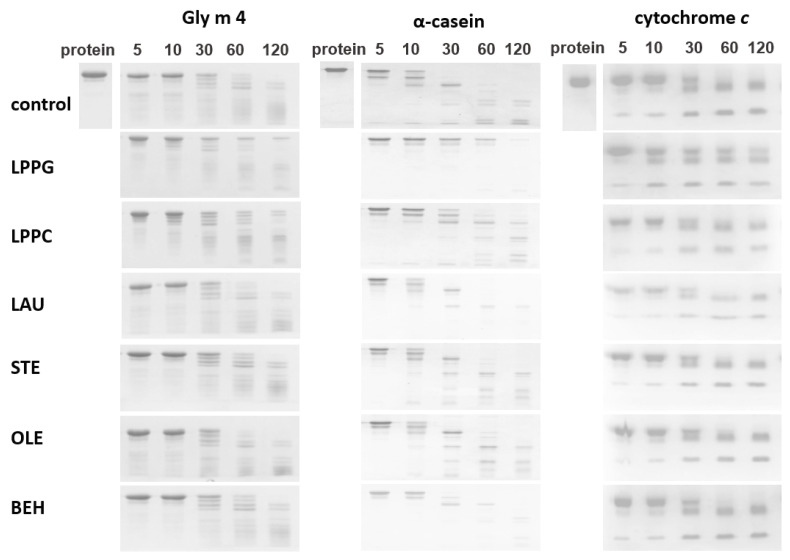
The effects of different lipids at concentrations of 0.2 mM on pepsin digestion of Gly m 4, α-casein or cytochrome *c*, at pH 2.0, as determined by SDS-PAGE (5, 10, 30, 60, and 120—digestion time, min). Protein—Gly m 4, α-casein or cytochrome *c* without pepsin; pepsin digestion was performed at the pepsin-to-substrate mass ratio of 1:2000 for Gly m 4 and for α-casein or of 1:10000 for cytochrome *c*.

The influence of selected FAs and lysolipids at concentrations of 0.2 or 0.02 mM on the rate of Gly m 4 pepsin digestion was examined (Figure 4 and Figure 5, Supplementary Figure S2). At pH 2.0 and pepsin-to-allergen mass ratio of 1:20, lipids did not affect Gly m 4 digestion, which proceeded very quickly under these conditions (data not shown). At the same pH value and pepsin-to-allergen mass ratio of 1:2000, the presence of LPPG and, to a lesser extent, LPPC at concentration of 0.2 mM (but not of 0.02 mM) reduced the rate of Gly m 4 digestion (Figure 5). In the presence of these lysolipids, approximately 25% and 10% of the intact Gly m 4, respectively, were found in the digests after 2 h, as compared with the control without lipids, in which the intact allergen was absent (Figure 4). The same effect was observed at pH 3.5 and pH 5.0 and at pepsin-to-allergen mass ratio of 1:200 (Figure 4). Only 29% and 65% of Gly m 4 were degraded after 2 h of pepsin digestion at pH 5.0 in the presence of LPPG and LPPC, respectively, compared with 82% in the control experiments without lipids (Figure 4). The investigation of the impacts of different lipids on pepsin digestion of model proteins, α-casein and cytochrome *c*, was carried out at pH 2.0 and at pepsin-to-protein mass ratio of 1:2000 or 1:10,000, respectively. LPPG and, to a lesser extent, LPPC at concentration of 0.2 mM slowed down α-casein degradation (Figure 5). Only LPPG reduced cytochrome *c* degradation under the above conditions.

### 3.4. Enzyme–Lipid Interactions

**Figure 6:**
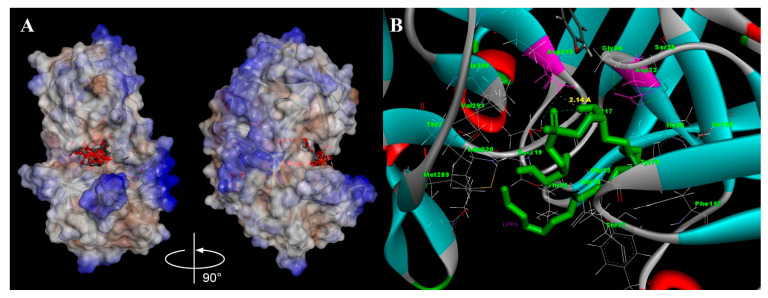
(**A**) Surface hydrophobicity of pepsin with 10 calculated conformations of LPPG: Asp32 and Asp215 are shown in red, hydrophilic amino acids are marked in blue, and hydrophobic amino acids are colored in brown. (**B**) Interaction of LPPG with the catalytic site of pepsin according to the molecular docking calculations: side chains of residues, contacting with the ligand, are shown with amino acid names and numbers; Asp32 and Asp215 are shown in magenta; hydrogen bonds are depicted as green dashed lines; hydrogen bond Asp215-LPPG (HO8 LPPG—Oδ1 Asp215) is shown in yellow.

The binding of different lipids with pepsin was studied by means of blind molecular docking. The AutoDock Vina software calculated 10 conformations of each lipid on the pepsin surface with different affinity energies. All of the studied lipids tended to be located at hydrophobic pocket where the catalytic site of the enzyme was localized (two aspartic acid residues, Asp32 and Asp215): 8 conformations of LPPG, 9 conformations of LAU and BEH, all 10 conformations of STE, OLE, and LPPC were identified at the catalytic site of pepsin. The pepsin spatial structure has several hydrophobic regions, and one of the largest hydrophobic moieties is located exactly at the catalytic site (Figure 6A). Both LPPC and LPPG had the highest affinity energies of −5.6 kcal mol^−1^ for the best conformations among all ligands. It was found that LPPG had the hydrogen bond with Asp215. In accordance with the accepted mechanism of pepsin-like enzyme function [27], Asp215 has to be negatively charged, whereas Asp32 has to be protonated. Hydrogen bonding of LPPG with Asp215 might be a reason for charge redistribution in the catalytic site of the enzyme and eventually be responsible for the inhibition of its enzymatic activity (Figure 6B).

## 4. Discussion

Stability of food allergens and their resistance to proteolysis are important factors that largely determine their allergenic potential. However, an intact true food allergen as well as its proteolytic fragments can induce sensitization of the immune system even if reaching the human intestine at microgram level [8]. It is known that Bet v 1 homologues are characterized by low stability and susceptibility to proteolysis, unlike lipid transfer proteins (LTPs) stabilized by disulfide bonds. But this statement is not related to all food allergens of this class. As believed, due to their low stability, most food Bet v 1 homologues cause mild cross-reactive allergic reactions in patients sensitized by the inhalant birch pollen allergen Bet v 1. However, some of them, such as the carrot allergen Dau c 1, the peanut allergen Ara h 8 [28], as well as the soybean allergen Gly m 4 [2,3], can induce systemic allergic reactions, including urticaria and anaphylaxis. In the present work, we studied the properties of the key soybean allergen Gly m 4 and the influence of various factors on its digestibility.

It is well known that food has different buffering capacity, and gastric conditions in fasted and fed states of stomach significantly differ from each other. In fed state, stomach pH can be increased up to values of 3–5 and pepsin-to-substrate ratio can be reduced. Moreover, normal pH as well as pepsin production are very different in adults, children under 7 years, and elderly people. For example, normal gastric pH values in the fasted state for adults or for children and elderly people are of 1–2 or 4–5, respectively [29,30]. In addition, conditions of gastric digestion can vary in people with chronic gastrointestinal diseases. In our previous study, we have shown a high susceptibility of Gly m 4 to gastric digestion, at pH 2.0 [15]. Here, the investigation of the effects of different pH values and pepsin-to-allergen ratios was performed to simulate non-optimal conditions for gastric digestion.

At first, the stability of the soybean allergen at different pH was studied using CD spectroscopy (Figure 2, Table S1). It was shown that Gly m 4, as well as the Bet v 1 homologues Mal d 1 from apple and Pru p 1 from peach [31], was denatured at pH 2.0, but not at pH 3.5 or at pH 5.0. Pepsin digestion of Gly m 4 under different conditions was carried out. A decrease in enzyme loading together with an increase in pH up to values of 3.5 and 5.0 led to significant reduction of the rate of allergen digestion (Figure 3). In this case, the intact soybean allergen was found in digests after 2 h. Probably, the slowing down of gastric digestion of Gly m 4 at more high pH values was associated not only with a decrease in pepsin activity, but also with the fact that, under these conditions, unlike at pH 2.0, the allergen had a secondary structure characteristic of Bet v 1 homologues in their native forms, and the sites for pepsin digestion were less accessible to enzyme. Thus, in the present study, we suggested that the Gly m 4 allergen is able to reach human intestine in its intact form.

Further, we investigated the ability of Gly m 4 to bind to different FAs and lysolipids at different pH values using TNS displacement experiments in vitro (Figure 1). In our previous work, we have shown that the lentil Len c 3, the allergen of the LTP class, bound to lipid ligands with almost identical affinity, at pH 7.4 and at pH 2.0 [32,33]. The present study aims to elucidate whether the selected lipids can be ligands of the soybean Bet v 1-like allergen as well as whether Gly m 4 can form complexes with these ligands under gastric conditions. We showed that, at neutral pH, the allergen with the highest effectiveness binds to OLE and LPPG, which are present not only in soybeans, but in many other plants and animal foods, and can be considered as possible ligands of Gly m 4. Surprisingly, it was revealed that the allergen was not able to bind these ligands not only at pH 2.0, but also at pH 3.5, at which, according to data of CD spectroscopy, the protein had the correct folding. A much lower affinity of the allergen for lipids was shown at pH 5.0, at which the protein binds only LPPG with moderate efficiency. We assumed that the Gly m 4 structure might vary with pH under acidic conditions and that additional methods instead of CD spectroscopy could shed some light. On the other hand, under acidic conditions, Gly m 4, with its low acidic pI, as well as lipids, have lower charge. Probably, charge-lowering affects the behavior of these molecules in solution and plays a role in initial protein–lipid interactions. We showed that OLE and LPPG are the probable ligands of Gly m 4. However, in the human stomach, this allergen can bind to only LPPG under weakly acidic conditions due to its sensitivity to pH changes.

Then, we investigated an influence of FAs and lysolipids on gastric digestion of Gly m 4 at pH 5.0, 3.5, and 2.0 values and revealed that LPPG, and, to a lesser extent, LPPC, at concentration of 0.2 mM, reduced the rate of pepsin digestion of the allergen (Figure 4 and Figure 5, Supplementary Figure S2). However, considering data on lipid binding at different pH values, we concluded that these effects were unlikely to result from the formation of the complexes of Gly m 4 with ligands. It is interesting to note that, at pH 2.0, LPPG also reduced the rate of pepsin digestion of α-casein and cytochrome *c*. These model proteins were used for comparison, as they were unstructured under these conditions, analogous to the soybean allergen, and did not bind to LPPG (Figure 2, Figure 4 and Figure 5, Table S1). An analysis of CD spectra revealed that, at pH 2.0, LPPG and LPPC, at the used concentration, affected the structures of Gly m 4 and model proteins increasing the percentage of α-helixes, but did not affect the structure of pepsin and did not cause denaturation of the enzyme. Lysolipids in solution at the used concentration of 0.2 mM could be present both as single molecules and in the form of micelles. Based on all of the obtained results, we suggested that the protective effect of lysolipids, especially LPPG, against pepsin digestion of all tested proteins, was associated either with the formation of micelles and the insertion of proteins into them or with a decrease in protein susceptibility to proteolysis due to a higher protein packing density, in the presence of lipids.

At the same time, it was shown that lipids can bind to digestive enzymes and inhibit their activity. For example, α-tocopherol in the steady-state fluorescence experiments bound to pepsin and inhibited its ability to cleave casein [34]. Here, we analyzed the pepsin spatial structure and revealed the presence of hydrophobic areas on its surface, one of which is located in the region of the catalytic site of the enzyme (Figure 6A). Lipid-binding experiments showed that pepsin is able to bind lipids, including LPPG, which displace the fluorescent probe TNS from hydrophobic regions of the surface of gastric enzyme (Figure 1). Computer modeling revealed that the most probable lipid-binding site in the pepsin molecule is located at a hydrophobic pocket nearby the catalytic site of the enzyme (Figure 6A). Among all of the tested ligands, lysolipids had the highest affinity energies, but only LPPG formed hydrogen bond with Asp215, constituting together with Asp32 the catalytic site of the aspartic protease (Figure 6B). Therefore, we suggested that a rate of the pepsin digestion of Gly m 4 and model proteins could be reduced also due to an ability of LPPG to bind and inhibit the activity of the gastric enzyme.

## 5. Conclusions

Previously, we have revealed that not only proteolytic fragments of Gly m 4, but also the intact soybean allergen is able to cross the Caco-2 epithelial barrier and induce the production of several anti- and proinflammatory stimuli by immunocompetent cells, which are responsible for Th2 response and sensitization of the immune system. It has been shown that Gly m 4, presenting in soybean seeds at relatively high level, is very susceptible to proteolysis under optimal gastric conditions. However, to date, it has not been demonstrated whether the allergen could reach the intestine in its intact form. In this study, we demonstrated that digestibility of Gly m 4 could be significantly reduced with the change of pH value and pepsin-to-allergen ratio, which are known to be critically dependent upon the amount and composition of ingested food. We showed that the presence of lipids, in particular, LPPG, may also facilitate this effect. LPPG is present in various foods and binds to Gly m 4 at different pH values. We hypothesized that LPPG can protect Gly m 4 against gastric digestion not by forming the protein-lipid complex, but by inserting the allergen in micelles and/or increasing the protein-packing density and/or inhibiting the pepsin proteolytic activity via interactions with its catalytic site. Thus, here, we described for the first time convincing arguments for the conclusion that at least some amount of the intact Gly m 4 allergen is able to reach human intestine under certain conditions and, in view of this, induce allergic sensitization.

## Data Availability

All data generated and analyzed during this study are included in this published article and its supplementary information file.

## Supplementary Materials

The following data are available online at https://www.mdpi.com/article/10.3390/membranes11100754/s1, Figure S1: SDS-PAGE analysis of stability of Gly m 4 in solutions with different pH values; Figure S2: The effects of different lipids at concentration of 0.2 mM on pepsin digestion of Gly m 4 at pH 3.5 and at pH 5.0; Table S1: Secondary structure estimation (%) predicted from far-UV CD spectra.
