# Supplementary material for: Do Lipids Influence Gastrointestinal Processing: A Case Study of Major Soybean Allergen Gly m 4
Source: Membranes (Basel). 2021 Sep 30;11(10):754. doi: 10.3390/membranes11100754 (PMC8537068; doi:10.3390/membranes11100754)
Supplement: Supplementary file 1 [file membranes-11-00754-s001.zip › Supporting information.pdf]

Supporting information

Table S1. Secondary structure estimation (%) predicted from far-UV CD spectra.

protein	conditions	α-helix, %	β-sheet, %	β-turn, %	random, %	NRMSD*
Gly m 4	pH 6.5	22.5	24.8	21.8	30.9	0.02
	pH 5.0	28.6	18.6	22.6	30.2	0.02
	pH 3.5	30.2	18.9	21.2	29.7	0.02
	pH 2.0	10.9	30.5	23.6	35.0	0.03
	pH 2.0+LPPG	18.8	25.0	22.5	33.7	0.01
	pH 2.0+LPPC	15.4	29.9	21.9	32.8	0.02
	pH 6.5+LPPG	29.3	20.9	20.8	28.9	0.02
	pH 6.5+LPPC	21.2	27.2	21.8	29.9	0.02
pepsin	pH 2.0	8.3	36.8	22.6	32.4	0.02
	pH 2.0+LPPG	9.6	35.3	22.7	32.4	0.04
	pH 2.0+LPPC	10.2	34.3	22.9	32.6	0.03
α-casein	pH 6.5	11.2	30.9	22.9	34.9	0.11
	pH 2.0	16.4	29.2	21.9	32.5	0.02
	pH 2.0+LPPG	14.5	33.1	22.3	30.1	0.04
	pH 2.0+LPPC	21.6	23.4	22.2	32.8	0.02
cytochrome *c*	pH 6.5	25.4	22.3	20.7	31.5	0.03
	pH 2.0	9.0	29.4	22.8	38.7	0.04
	pH 2.0+LPPG	17.1	28.1	22.0	32.7	0.04
	pH 2.0+LPPC	11.6	30.2	22.6	35.7	0.03

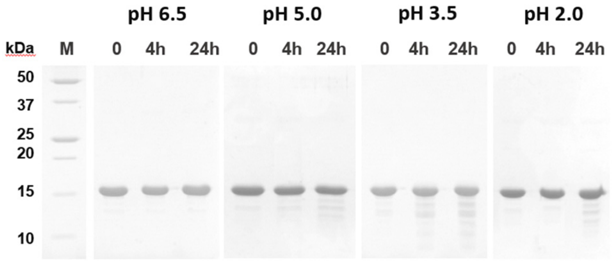

Figure S1. SDS-PAGE analysis of stability of Gly m 4 in solutions with different pH values: M – molecular mass standards; 0, 4h, 24h – aliquots of the protein solutions in initial moment and after incubation during 4 h and 24 h, respectively.

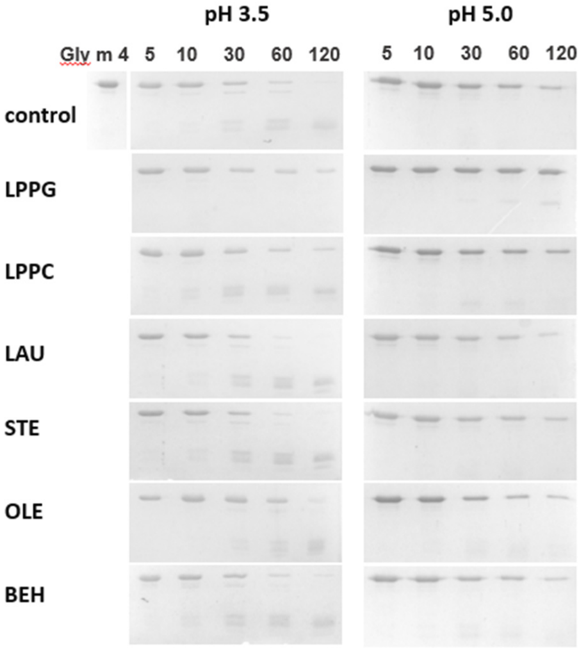

Figure S2. Effects of different lipids at concentrations of 0.2 mM on pepsin digestion of Gly m 4 at pH 3.5 and at pH 5.0, as determined by SDS-PAGE (5, 10, 30, 60, 120 – digestion time, min). Pepsin digestion was performed at pepsin-to-substrate mass ratio of 1:200.
